# Going the Distance for Protein Function Prediction: A New Distance Metric for Protein Interaction Networks

**DOI:** 10.1371/journal.pone.0076339

**Published:** 2013-10-23

**Authors:** Mengfei Cao, Hao Zhang, Jisoo Park, Noah M. Daniels, Mark E. Crovella, Lenore J. Cowen, Benjamin Hescott

**Affiliations:** 1 Department of Computer Science, Tufts University, Medford, Massachusetts, United States of America; 2 Department of Computer Science, Boston University, Boston, Massachusetts, United States of America; Technical University of Madrid, Italy

## Abstract

In protein-protein interaction (PPI) networks, functional similarity is often inferred based on the function of directly interacting proteins, or more generally, some notion of interaction network proximity among proteins in a local neighborhood. Prior methods typically measure proximity as the shortest-path distance in the network, but this has only a limited ability to capture fine-grained neighborhood distinctions, because most proteins are close to each other, and there are many ties in proximity. We introduce diffusion state distance (DSD), a new metric based on a graph diffusion property, designed to capture finer-grained distinctions in proximity for transfer of functional annotation in PPI networks. We present a tool that, when input a PPI network, will output the DSD distances between every pair of proteins. We show that replacing the shortest-path metric by DSD improves the performance of classical function prediction methods across the board.

## Introduction

One of the best-studied classical problems on biological networks involves using proteins of known function, together with the structure of the network of known protein-protein interactions (PPI) to make predictions of functions of unlabeled protein nodes. This is an important problem because, even in the best-studied model organisms, such as *S. cerevisiae*, these networks contain many proteins whose function is still completely uncharacterized. There are many proposed methods for this problem, including versions of majority voting [1], neighborhood algorithms [2], [3], clustering algorithms [4]–[6], algorithms based on maximum flow [7], or multi-way cut [8], [9], and a number of others [10].

Modern approaches also seek to deal with data quality: generally, the known network is missing many valid interactions, and some interactions may be known with higher confidence than others [11], [12]. Other modern approaches integrate PPI network data with high-throughput biological interaction data, such as sequence information, genetic interactions, structural information, and expression data [8], [13]–[15]. However, nearly all methods that predict function using PPI network structure depend, entirely or at least partially, on the simple shortest-path distance metric applied to the PPI graph.

We start with the observation that there is an intrinsic drawback to relying on the ordinary shortest-path distance metric in PPI networks. PPI networks are known to be “small world” networks in the sense that they are small-diameter, and most nodes are close to all other nodes (though the exact details of the degree distribution and the resulting extent to which they are “scale free” is a subject of lively debate, see [13], [16]–[19]). Thus any method that infers similarity based on proximity will find that a large fraction of the network is proximate to any typical node. In fact, this issue has already been termed the “ties in proximity” problem in the computational biology literature [4].

Furthermore, the fact that two nodes are adjacent (i.e., have shortest-path distance 1) in a PPI network can signify something very different than the adjacency of two other nodes. For example, as we discuss below, in PPI networks two nodes with many low-degree neighbors in common should be thought of as “more similar” than nodes with few low-degree neighbors in common; and such nodes should also be thought of as “more similar” than two nodes whose common neighbors have high degree. Thus, characterizing node pairs based only on a shortest-path notion of distance fails to capture important knowledge encoded in the structure of the network.

What is needed instead is a finer-grained distance metric, capable of making more subtle distinctions of similarity than ordinary shortest-path distance would in a small-world network. To that end we introduce a new metric called *Diffusion State Distance*, or DSD. We show that DSD is much more effective than shortest-path distance for the problem of transferring functional labels across nodes in PPI networks.

We demonstrate the utility of the DSD metric by modifying a number of the most popular classical algorithms for function prediction to replace the ordinary notion of distance with DSD. For the problem of predicting functional labels in the *S. cerevisiae* network, this change improves *all* the algorithms we consider. We then show that similar improvements hold for the more sparsely annotated *S. pombe* network, implying that our advances should generalize to other biological networks.

### Motivation for DSD

To start, we consider all known physical interactions in the *S. cerevisiae* PPI network – specifically, version 3.2.102 of BioGRID [20] on verified ORFs for *S. cerevisiae*, which contains 128,643 annotated physical interactions. After removing redundant edges and selecting the largest connected component, the resulting PPI network has 4990 nodes (where each ORF corresponds to a node) and 74,310 edges (where each edge corresponds to an annotated physical interaction).

**Figure 1:**
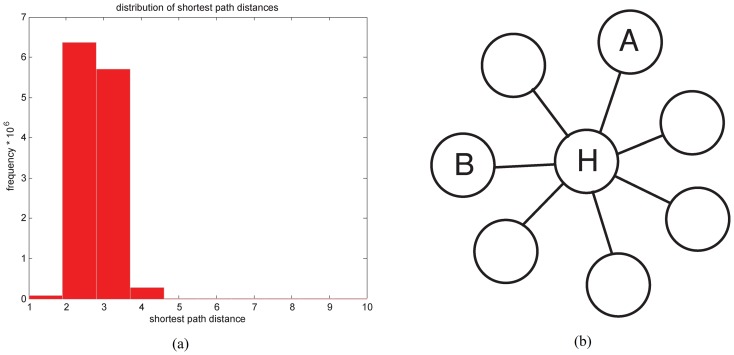
Structure of shortest paths in the yeast PPI network. (a) Distribution of shortest-path distances in the largest connected component of the yeast PPI network; (b) an example subgraph with a hub.

Figure 1(a) shows the histogram of shortest-path lengths from this network. The figure shows that almost all pairs of nodes (over 95%) are either 2 hops or 3 hops apart. Thus the “typical” distance between nodes under this metric is very small. Likewise, the overall network diameter is quite small as well.

The fact that most node pairs are quite close together means that the concept of “neighborhood” using this metric is not very useful. For example, if one looks at the 2-hop neighborhood of a typical node, it probably includes around half of all nodes in the graph. Hence, shortest-path distance cannot be expected to give a good sense of locality or modularity in this network, and we observe that if one seeks to use graph structure to infer similarity of function in a PPI network, using shortest-paths to measure distance has serious drawbacks.

One of the reasons that paths are typically short in biological networks like the PPI network is due to the presence of *hubs* – very high degree nodes which represent proteins that have many physical interaction partners. In fact, in the case of PPI networks, hubs often represent proteins with *different* functional roles than their neighbors. For example, chaperone proteins (proteins that help other proteins fold correctly) are often hubs, and are not typically functionally related to their interaction partners. The same is true for proteins involved in translation. In our network, as an extreme example, the highest degree node is the protein NAB2, which has 2325 physical interactions in our PPI network. Its functional annotation terms: “3′-end processing”, “RNA binding” and “RNA transport” [21] suggest that this protein is involved in translation machinery and thus will bind with a highly diverse set of proteins with unrelated function. Hubs are also more likely to be proteins with multiple, distinct functions [22].

Hence, not all short paths provide equally strong evidence of similar function in PPI networks. Consider the network in Figure 1(b). Although nodes 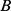 and 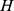 are only one hop apart, this does not suggest they are functionally related, since 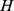 is a hub. Likewise, 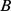 and 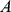 are not necessarily functionally related, since they are connected through a hub.

To capture the notion that 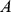 and 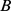 are not necessarily related, we note that a random walk starting at 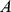 is not likely to reach 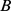 quickly. If we restrict attention to random walks of, say, 3 hops, then often one will not reach 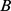 from 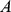 at all.

This motivates the first element of our new metric definition. Given some fixed 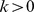, we define 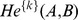 to be the expected number of times that a random walk starting at 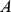 and proceeding for 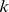 steps, will visit 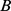. Note that 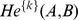 bears some resemblance to previous diffusion approaches suggested for PPI networks, see [23], [24], though we will next be building metric structure on top of 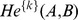 in a completely novel way. For now, note that 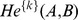 captures our intuition regarding similarity, because node pairs connected by many short paths of low-degree nodes will tend to have high 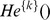 values. If node pairs with a large 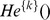 value are then somehow considered ‘similar’ in our metric, then clearly 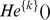 does a better job of capturing ‘similarity’ than does shortest-path distance. This is because 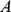 and 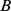 are relatively far under this metric; the influence of the hub 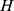 decreases the likelihood of a random walk from 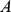 landing at 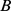.

But a metric whose notion of similarity is based only on using 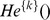 between the two given nodes directly does not solve all our problems. In particular, note that 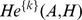 will indicate strong similarity, even though (as we have argued) 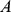 is not likely to be strongly functionally related to 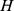. Furthermore, 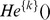 is still far from a metric; note it is not even symmetric. Also, 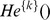 is only a pairwise, and not a global measure of node similarity. This observation leads us to use 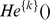 in a more subtle way, and in a different manner than previous diffusion approaches, resulting in the definition of DSD, which we describe next.

### Definition of the New Distance Metric

Consider the undirected graph 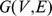 on the vertex set 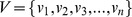 and 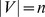. Recall that 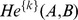 is defined as the expected number of times that a random walk starting at node 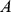 and proceeding for 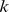 steps, will visit node 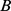. In what follows, assume 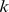 is fixed, and when there is no ambiguity, in the value of 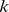, we will denote 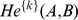 by 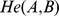. We further define a 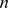-dimensional vector 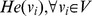, where
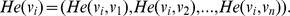
Then, the Diffusion State Distance (DSD) between two vertices 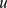 and 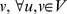 is defined as:



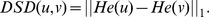
where 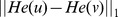 denotes the 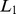 norm of the He vectors of u and v.

We will show for any fixed 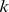, that DSD is a metric, namely that it is symmetric, positive definite, and non-zero whenever 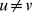, and it obeys the triangle inequality. Thus, one can use DSD to reason about distances in a network in a sound manner. Further, we show that DSD converges as the 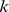 in 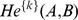 goes to infinity, allowing us to define DSD independent from the value 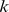.

### Characteristics of DSD

**Figure 2:**
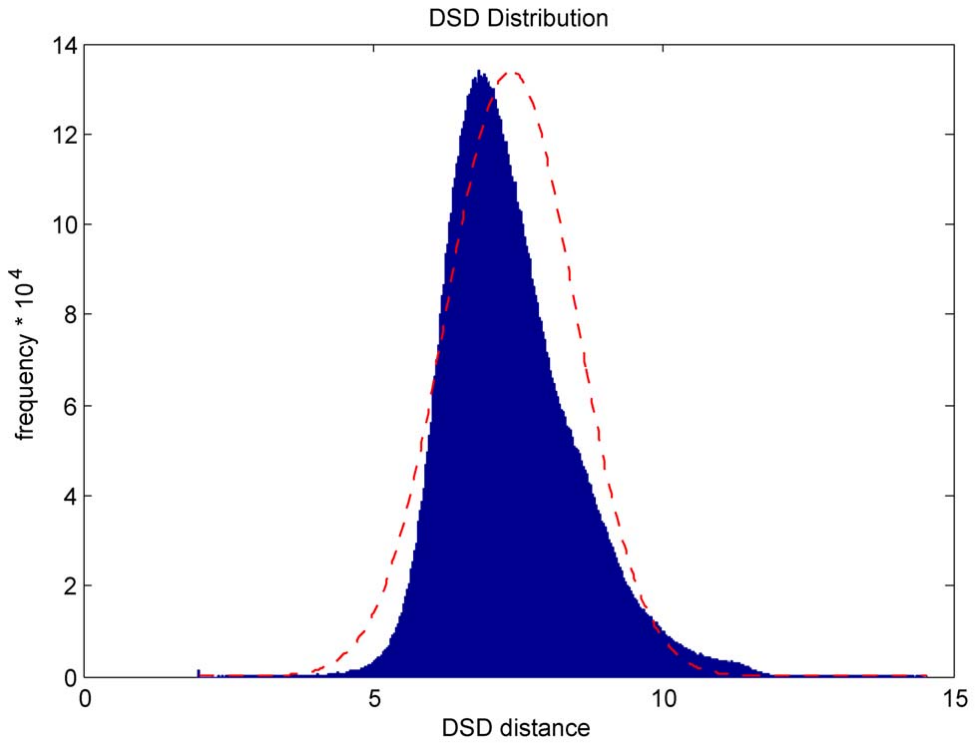
Distribution of DSD in the largest connected component of the yeast PPI network; the red curve represents a fitted normal distribution for comparison.

Figure 2 shows the distribution of DSD values in the PPI network of *S. cerevisiae* as downloaded in BioGRID [20]. The figure shows that DSD distances take on a smooth, continuous range of values. This is in sharp contrast to shortest-path distances, as shown in Figure 1(a), and shows that DSD is capable of making much finer-grained distinctions of similarity over pairs of nodes in the network.

**Figure 3:**
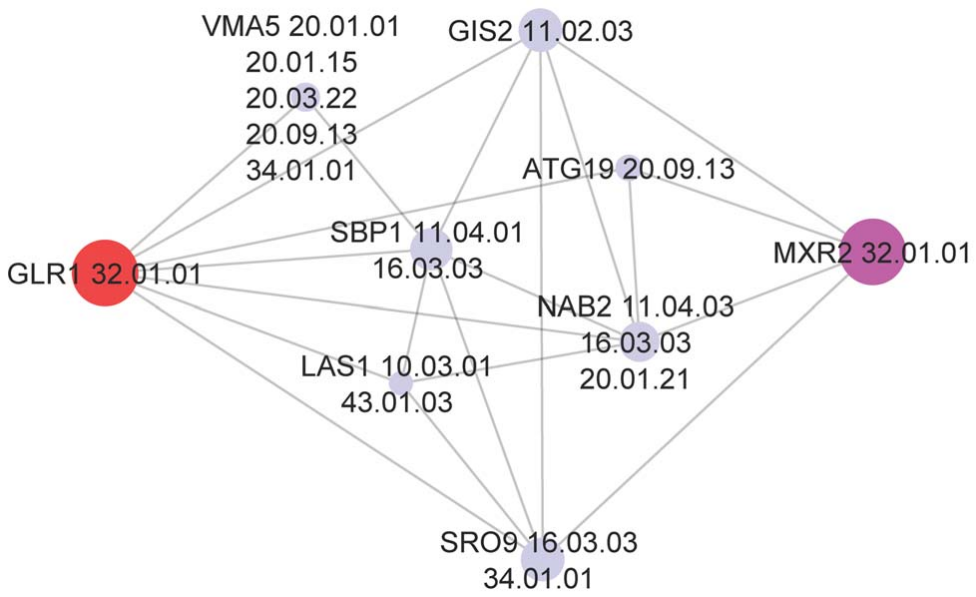
An example of functional annotation with DSD. The correct functional annotation for GLR1, on the third level of the MIPS hierarchy, 32.01.01 (oxidative stress response) is found among none of its direct neighbors, but with the node that is closest in DSD, MXR2. MXR2 is closest in DSD because it has the most similar neighborhood to GLR1.

Figure 3 shows a typical example illustrating the nature of DSD. The Figure shows the gene GLR1 (in red at left) along with a subset of its local neighborhood. Node sizes have been made inversely proportional to their DSD distance from GLR1. None of GLR1's immediate neighbors contain its correct functional label at the third level of the MIPS hierarchy (32.01.01: oxidative stress response). However, the node closest in DSD is MXR2, which has exactly the same functional label. DSD recognizes that nodes having large common low-degree neighborhoods are highly similar and correctly identifies functionally similar node pairs, and does so in situations where shortest-path distance fails.

## Methods

### Functional Categories

We continue to work with the dataset described above, the largest connected component from an experimentally derived physical interaction PPI network from *S. cerevisiae* with 4990 nodes, and 74,310 edges. As in the papers introducing the classical function prediction methods we consider, we primarily use the MIPS (Munich Information Center For Protein Sequences) functional catalogue (FunCat) for our functional category labels [21]. We use the latest version of FunCat (version 2.1) and the first, second and third level functional categories, retaining those for which at least three proteins in our dataset are annotated with those labels. We present results for the first level (17 functional categories) second level (74 of the 80 functional categories that annotate at least one protein, annotate at least 3 proteins) and third level (154 of 181 functional categories that annotate at least one protein, annotate at least 3 proteins) MIPS annotations. MIPS is a shallow, leveled, hierarchical classification scheme for protein function, but we also present results for the popular Gene Ontology (GO) [25], where the variable depth hierarchy of the annotation labels makes the evaluation of labeling methods more complicated.

We assumed all published labels were correct and did not attempt for this study to weigh them by a level of confidence in each type of biological experiment. The following classical methods were tested in their original form (using shortest-path distance), against a DSD variant in cross validation.

### Cross Validation Task

We considered 2-fold cross validation tasks. In each of the 2-fold cross validation tasks, we first randomly split the annotated proteins into 2 sets, and consider only the annotations on one partition in the training set, when trying to predict the annotations on proteins in the test set, and average the performance over the 2 folds of the cross validation. We conduct 10 runs of 2-fold cross-validation and report the mean and standard deviation of the following performance measures over these 10 runs.

#### Accuracy

This is the performance measurement suggested in [1]. Each protein is assigned its highest-scoring functional label. The label is considered correct if it appears among the labels that were assigned to the protein. We calculate the percentage of proteins that are assigned a correct label.

#### F1 score

This is the performance measurement suggested in [26]. For each protein, the possible functional labels the algorithm could assign are stored in a ranked list according to score. Each label is considered correct if it appears among the labels that were assigned to the protein, and incorrect otherwise. We calculate precision and recall by looking at the top 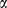 (in our case, we present results for 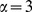) predicted annotations. Then the F1 score for each function can be calculated as:
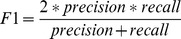



We average F1 scores over the individual functions and obtain the overall F1 score for each algorithm.

### Protein Function Prediction Methods

#### Neighborhood Majority Voting Algorithm

This is the simplest of all function prediction methods. Directly applying the concept of ‘guilt by association’, Schwikowski et al. [1] considered for each protein 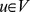 its neighboring proteins. Each neighbor votes for their own annotations, and the majority is used as the predicted functional label. To incorporate DSD, the neighborhood of 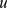 is defined simply as the 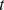 nearest neighbors of 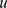 under the DSD metric. Furthermore, two schemes are considered: an unweighted scheme where all new neighbors vote equally, and a DSD weighted scheme where all new neighbors get a vote proportional to the reciprocal of their DSD distance.

#### 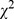 Neighborhood Algorithm

In the original 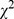 neighborhood algorithm [3], each annotation 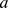 present in protein 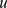
's neighborhood will be assigned a 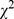 value based on the following formula:
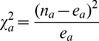
where 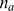 is the observed number of annotations 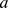 in the neighborhood, and 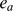 is the expected number of annotations 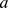 based on the whole protein-protein interaction network. Then protein 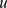 is assigned the functional label 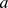 with the maximum 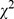 value. Again, it is straightforward to adapt this to use DSD: the neighborhood of 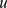 is simply defined as the 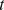 closest nodes to 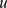 under the DSD metric.

#### Multi-way cut Algorithm

We consider the minimal multi-way k-cut algorithm of Vazquez et al. [9] as implemented by [7]. The motivation is to minimize the number of times that annotations associated with neighboring proteins differ. In particular, the dual ILP (integer linear programming) is formulated, so we instead seek to maximize
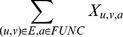



subject to the constraints 
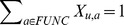
, 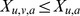, 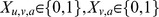 where the edge variables 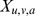 are defined for each function 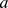 whenever there exists an edge between proteins 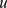 and 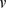. It is set to 1, if protein 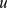 and 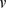 both are assigned function 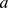, and 0 otherwise. The node variables 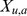 are set to 1 when 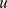 is labeled with function 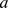 and 0 otherwise. The first constraint insures that each protein is only given one annotation. The second constraint makes sure only annotations that appear among the vertices can be assigned to the edges. While this problem is NP-hard, the ILP is tractable in practice; in our case we use the IBM CPLEX solver (version 12.4, dated 12/2011, http://www.ilog.com/products/cplex/). For the DSD version, we simply add additional edges between vertices whose DSD is below a threshold. We set a global threshold 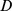 based on the average DSD of all pairs, specifically we set 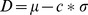, where 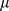 is the average, and 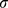 is the standard deviation of the global set of DSD values among all pairs of nodes in the graph. We experiment with 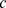 in the range 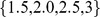.

#### Functional Flow Algorithm

Nabieva at al. [7] use a network flow algorithm on the graph of protein interactions to label proteins. The idea is to consider each protein having a known function annotation as a ‘reservoir’ of that function, and to simulate flow of functional association through the network to make predictions. We adapt the approach to use DSD by creating an edge between each node pair, with a weight inversely proportional to DSD. For computational efficiency we do not create edges when the reciprocal of DSD is below a small value. As in the original functional flow, we calculate flow through this new network at each time step. We denote the size of the reservoir of function 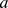 at node 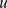 and time step 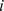, to be 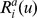. For a given function (annotation) 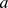, we initialize the reservoir size at node 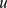 to be infinite if protein 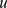 has been annotated with function 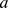; otherwise we set it to be 0. More formally:
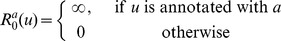



We then update the reservoir over a sequence of timesteps (we use 6 timesteps, as in the original version):
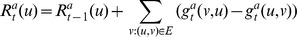
where 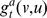 is the amount of flow 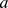 that moves from 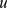 to 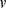 at time 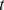. We incorporate DSD into the edge weight as follows:



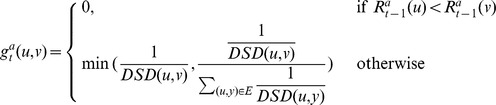



The final functional score for node 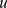 and function 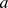 over 6 timesteps is computed as the total amount of incoming flow.

### Formal Properties of DSD

We now present the formal proofs that DSD is a metric for any fixed 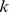, that it converges as 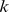 goes to infinity, and obtain the explicit form that allows the calculation of DSD values in the limit.

#### Lemma 1


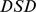
 is a metric on 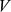, where 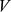 is the vertex set of a simple connected graph 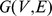.

#### Proof

Clearly the DSD of a node to itself is 0, and DSD is non-negative and symmetric. It remains only to show that DSD satisfies the triangle inequality, and that 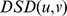 is strictly positive whenever 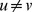.

For all 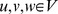, we have by the 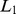 norm property:
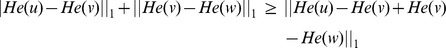



and therefore:
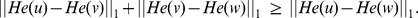



Thus, the triangle inequality follows easily:
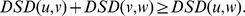



Next we prove the *identity of indiscernibles*, namely, 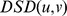 is non-zero for all 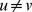. We first need some notation. We define the one step transition probability matrix 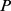 as the 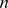-dimensional square matrix, whose 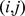
th entry is given by:
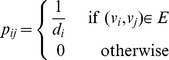
where 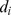 is the degree of node 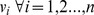. Note that 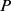 represents the probability to reach each neighbor in the random walk where all neighbors are reached with equal probability.

Then the definition of 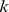 step transition probability matrix 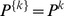 follows for all positive 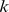. We also define the 0 step transition matrix 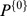 to be the identity matrix 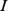, because every random walk starts from the source vertex, and thus a zero-step random walk reaches its source vertex with probability 1 and all other vertices with probability 0. We denote by 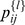 or 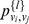 the 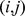
th entry in the 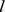
th transition probability matrix, where 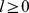 and 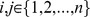.

For each ordered pair of vertices 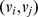, recall that 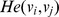 is defined as the expected number of times that a random walk with length 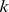 starting from 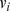 will visit 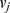. In order to calculate 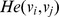, we define an indicator variable 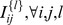, where:
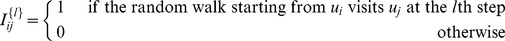



Therefore we have 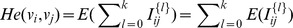 by linearity of expectation. Clearly, 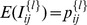, and thus we have 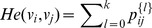. Note that because we are adding the zero-step transition matrix, we will have that 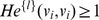, 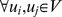.

Now we are ready to show the *identity of indiscernibles*. Recall we are assuming the graph is connected. It is trivial when 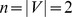, so consider the case where 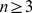. We prove this next by contradition.

Assume there exists a pair of distinct vertices 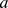 and 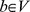 in the simple connected graph G, where 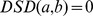. Thus 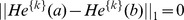, and 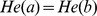, which indicates that 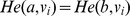, 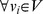. We have 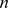 equations now: 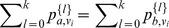. Since the zero-step transition matrix 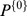 is an identity matrix, we know that 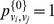 for 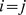 and 0, otherwise.

Thus we have
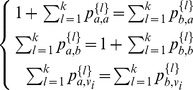
(1)where the third line represents a set of 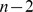 equations: one for each 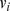, distinct from 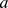 and 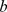.

By multiplying a factor 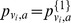 to both sides of all equations in the third line and summing over 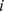, we have:
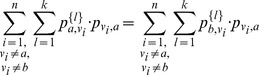



By completing the sum over 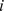, we have:
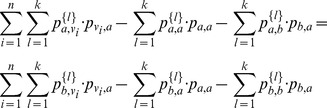



G doesn't contain self-loops; thus 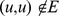 and 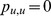, 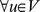. For each 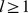, we have
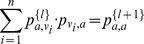



and
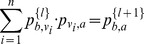



Therefore, we have
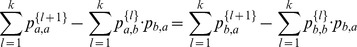



namely,
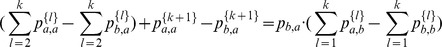



By applying to the equation above the first and the second equation in (1), where:
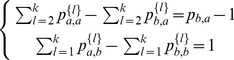
(2)


we have:
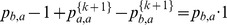



namely,
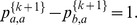
Since 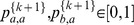, we have 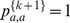.

We next argue that it must be the case that 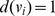, for all 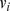 with 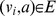, namely, all of 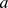
's neighbors must have degree 1.

Starting from
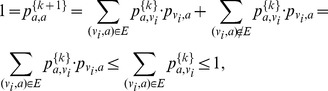



we must therefore have 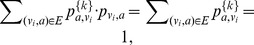 and 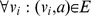 and 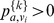, 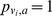. Since we have 
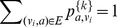
, there must exist at least one neighbor 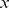, s.t. 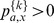, and 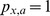 as well. Because 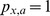, we know that 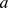 is the only neighbor to 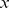 and 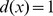. Also due to the fact that 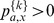, there exists a path of length 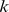 from 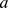 to 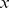, which should consists of two parts, one path (denoted as 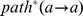) of length 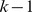 from 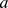 to 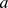 and an edge from 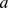 to 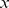 as the last step in the path.

Consider any neighbor 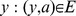. By using the 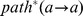 and the edge 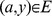, we can construct a path of length 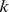 from 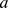 to 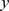 and therefore 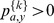. Thus, 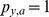, and therefore 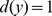. Thus we have shown that it must be the case that 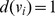, for all 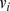, with 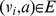.

As a result, all of 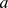
's neighbors are only connected to 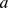.

If 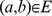, then 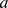 and 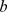 can't have any more neighbors because they must be the only neighbor of each other; thus for all 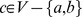, 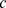 is not connected to 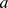 or 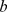, which contradicts the fact that the graph G is connected. If 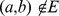, 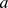 and 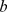 are not connected because all of their neighbors are only adjacent to themselves respectively.

Therefore, the existence of such pair 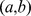 where 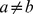 and 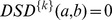 contradicts the fact that the graph is connected, and we can conclude that 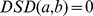 if and only if 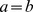.

In the above we were reasoning about DSD for a fixed value of 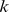. Denote by 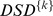 the value of DSD for a particular fixed 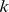. Next we discuss how DSD values depend on 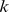. We show that when the one-step transition matrix 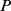 is diagonalizable then 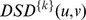 converges to a stationary value 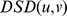.

#### Lemma 2

Let 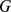 be a connected graph whose random walk one-step transition probability matrix 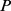 is diagonalizable and ergodic as a Markov chain, then for any 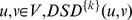 converges as 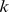, the length of the random walk, approaches infinity.

#### Proof

Since the one step transition probability matrix 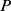 is diagonalizable, we have by diagonalization:
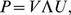
where the orthogonal matrix 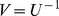; each column of 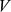 is the normalized right eigenvector of 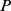, each row of 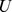 is the normalized left eigenvector of 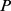, and 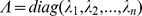 is the diagonal matrix of all eigenvalues where 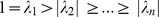 by ergodicity. Let 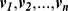 denote the normalized orthogonal column vectors in 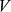 and 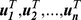 the normalized orthogonal row vectors in 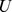. We denote 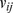 as the 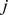
th entry in vector 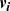 and 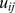 as the jth entry in vector 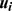.

Thus it follows that 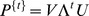, namely 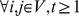:
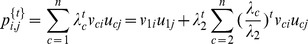



Next, for all 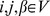, we define an infinite sequence 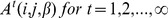:
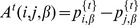


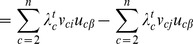


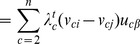



and 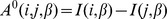 where 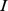 is the identity matrix.

Therefore, by definition, we can rewrite 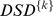 as the partial sum:
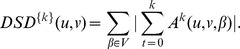



#### Claim 1

For all 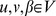, 
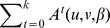
 converges absolutely.

Before we prove the claim, we show that it produces what we need. If the claim is true, then the limit 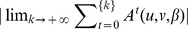 exists, and we can denote it as 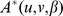. Then it follows that:
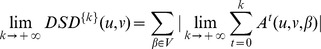


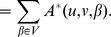



and we have proved convergence.

But Claim 1 is true from the Cauchy root test, namely,
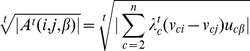
(3)

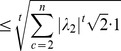
(4)

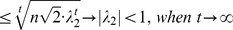
(5)where (3) & (5) hold by definition and (4) holds because 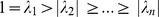 and 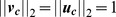.

We now use this lemma to produce an explicit way to calculate in the limit. This frees DSD entirely from dependence on the parameter 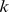, and this is the version of DSD we use in our experiments. (We remark, that for our yeast PPI network, we found empirically that 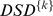 values were very close to the limit when 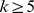.)

#### Lemma 3

Let 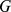 be a connected graph whose random walk one-step transition probability matrix 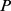 is diagonalizable and ergodic as a Markov chain, then for any 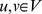, we have 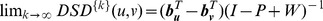, where 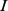 is the identity matrix, 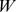 is the constant matrix in which each row is a copy of 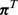, 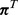 is the unique steady state distribution, and for any 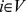, 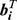 is the 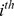 basis vector, i.e., the row vector of all zeros except for a 1 in the 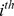 position.

#### Proof

To start, we denote the matrix 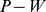 by 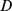. By Lemma 2 where we have shown that 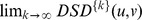 exists and is finite, we can denote the limit as simply 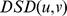 in the following context. It follows that for any 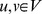:
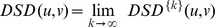


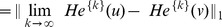


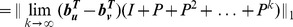
(6)

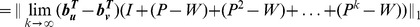
(7)

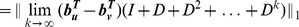
(8)

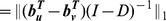
(9)where (6) holds by definition, (7) holds because 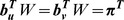, (8) holds because 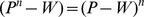 for all 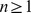 by Claim 2, and (9) holds by Claim 3, where we finish the proof of Lemma 3 by proving Claims 2 and 3 next.

#### Claim 2


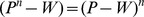
, for any non-zero positive integer n.

#### Proof

The proof is based on the following observations: 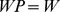 and 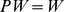, so 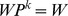 and 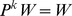. Further, 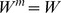, so 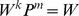 and 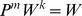 for any integers 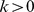 and 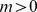. Now, if you construct the binomial expansion of 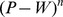, each cross term of the type 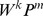 or 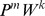 reduces to 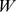, and all of the 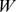
s cancel out except for one, leaving 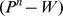. Thus, 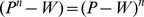.

#### Claim 3


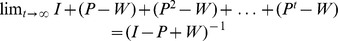
.

#### Proof

By Claim 2, 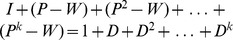

1. First we show that 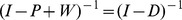 exists. We show that 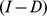 has full rank by showing that 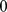 is the only vector in the left nullspace of 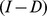. That is, if 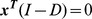, then 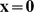.

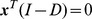


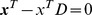


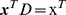


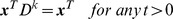



Now, we already know that 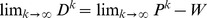 exists and is 0 by ergodicity. So we have
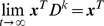


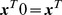



which is only satisfied by by 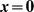.

1. Since 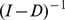 exists and 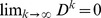, we can calculate as follows:

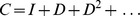


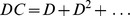


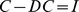


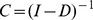
where 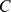 is defined as 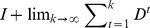, which exists because 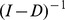 exists and 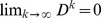.

That finishes the proof for Lemma 3. 

## Results

### MIPS Results

**Figure 4:**
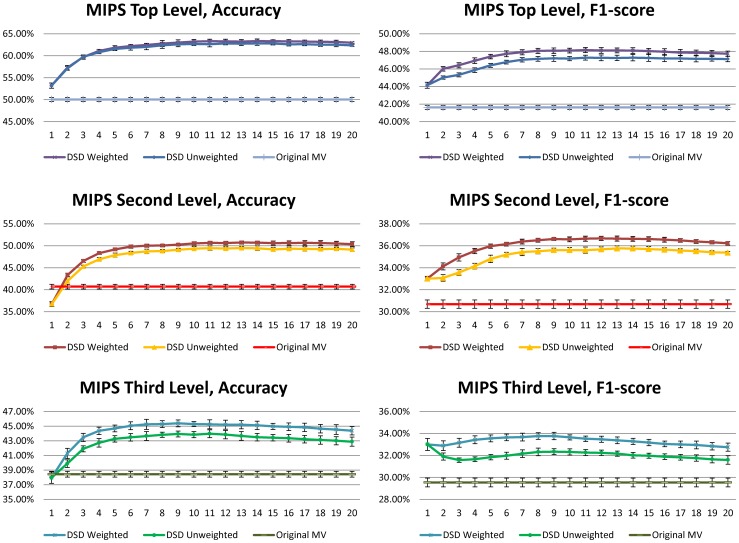
Improvement on Accuracy and F1 Score for at different neighborhood thresholds, for the majority voting algorithm in 10 runs of 2-fold cross validation for *S. cerevisiae*, with standard deviations.

**Figure 5:**
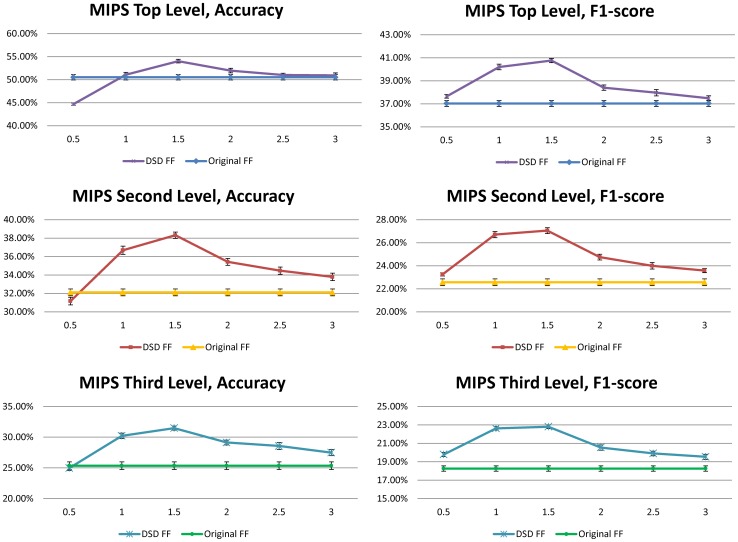
Improvement on Accuracy and F1 Score for DSD at different neighborhood thresholds, for the functional flow (FF) algorithm in 10 runs of 2-fold cross validation for *S. cerevisiae*, with standard deviations.

Both the DSD majority voting method and the 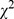 neighborhood method sort vertices in order of their smallest DSD to a given vertex, and set a threshold 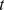, where the first 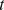 vertices participate in the functional vote. Table 1 gives the results in 2-fold cross validation for both these methods when we set 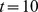, whereas Figure 4 gives more details about the dependence on 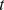; basically once enough neighbors were included (i.e. 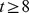) results of the DSD version of majority voting converged. Similar details about how the 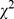 neighborhood method depends on 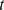 appears in (Figure S1). For the multi-way cut and functional flow results, we considered vertices to be in the DSD neighborhood of a node 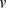 if their DSD from 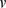 was less than 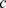 standard deviations below the mean DSD value across the entire dataset. Table 1 presents the 2-fold cross validation DSD multi-way cut results and DSD functional flow results with 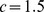. In addition, Figure 5 gives more detail about the dependence of DSD functional flow on the parameter 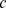, where we tested 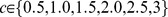. Similar details about how the multiway cut method depends on 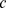 appear in (Figure S2).

**Table 1:** Summary of DSD improvements of all four methods in 2-fold cross validation (mean and standard deviation in percentage) for the PPI network of *S. cerevisiae.*

	MIPS 1		MIPS 2		MIPS 3	
	Accuracy	F1 score	Accuracy	F1 score	Accuracy	F1
Majority Vote (MV)	50.0±0.5	41.6±0.2	40.7±0.5	30.7±0.4	38.4±0.4	29.5±0.4
MV with DSD	63.7±0.4	47.2±0.2	49.3±0.5	35.6±0.2	43.8±0.4	32.3±0.3
MV (weighted DSD)	63.2±0.5	48.1±0.3	50.6±0.4	36.6±0.2	45.3±0.3	33.6±0.2
Neighborhood (NH)	43.3±0.3	34.5±0.2	32.4±0.6	26.1±0.3	31.3±0.5	24.8±0.3
NH with DSD	51.5±0.4	40.6±0.3	34.8±0.5	27.7±0.2	32.6±0.6	25.1±0.3
Multi-cut	55.2±0.4	42.1±0.2	42.0±0.6	28.1±0.2	36.6±0.4	24.8±0.3
Multi-cut with DSD	58.3±0.3	42.2±0.2	44.6±0.4	29.6±0.1	38.2±0.3	25.3±0.2
Functional Flow (FF)	50.5±0.6	37.0±0.3	32.1±0.4	22.6±0.3	25.4±0.6	18.3±0.3
FF with DSD	54.0±0.4	40.8±0.2	38.3±0.3	27.1±0.3	31.5±0.3	22.8±0.2

We notice from Table 1 that in every case, the DSD versions of all four methods perform better than the versions based on ordinary distance. It is particularly interesting that using DSD improves the functional flow algorithm, since functional flow already seeks to capture a notion of diffusion in the graph. In fact, the DSD versions of Majority Voting perform better than all four of the methods based on ordinary distance. The best performing algorithms among all the ordinary distance algorithms on the yeast MIPS annotations at all three levels of the MIPS hierarchy were the majority voting algorithms. The best performing algorithms overall are the DSD version of the majority voting algorithms, and they clearly achieve the best performance compared to all four ordinary distance and all three other DSD-based methods. In particular, they achieve 63.7% mean accuracy and 47.2% mean F1 score (unweighted) and 63.2% mean accuracy and 48.1% mean F1 score (weighted) on the first level of the MIPS hierarchy. This is compared to the original majority voting algorithm, which only obtains 50.0% mean accuracy and 41.6% mean F1 score. Hence DSD provides more than 13% improvement in accuracy and more than 5% improvement in F1 score for MIPS top level functional categories, and improves on ordinary DSD on the second and third levels of the MIPS hierarchy across the board.

### GO Results

GO (Gene Ontology) [25] is a deeply hierarchical classification scheme, which makes defining and evaluating function prediction methods much more complicated. For this reason, most function prediction methods for yeast PPI networks use the “flatter” set of MIPS categories, but GO is the more widely used ontology. Thus we wished to measure performance for GO functional labels, despite the difficulties. For example, in GO, sometimes, nodes are labeled with child functions but not labeled with their parent functions, even though it is assumed that child functions are more specific and inherit the functions of their parent nodes. How much credit should be given to a node when we do not label it correctly at its most specific level, but succeed in labeling it with a less specific ancestor term? The exact match and functional path methods of Deng et al [27], [28] are designed to directly answer this question and evaluate methods that perform GO annotation. We tested the performance of ordinary distance versus DSD versions all four protein function prediction methods considered above also on the GO, and evaluated the results using the exact match method and functional path method of Deng et al. [27]. We find, again, that using the DSD-based algorithm improves performance.

We consider labels in the biological process category of the GO hierarchy (we used OBO v1.2 [29], data version 2013-07-18) along with annotations downloaded from the SGD database (data version 2013-07-13). We exclude GO terms that are annotated with evidence codes “IEA” “RCA” or “IPI”. For each protein that was labeled with a term in the GO hierarchy, we automatically also label it with all more general parent terms in the GO hierarchy as well. We then, define the *informative* nodes in the GO hierarchy to be functional annotation terms that 1) are at least three levels below the root and 2) are terms that annotate more than 50 proteins in our dataset, where the second condition on informative nodes, and the number 50 is suggested by the method of Deng et al. [27]. It is these GO functional terms, somehow capturing the middle levels in functional specificity, that we use instead of a level of the MIPS hierarchy for the functional labels. The result is 136 informative biological process GO terms among 4322 out of 4990 ORFs that will comprise our labeling set (the total number of annotations is 58,519); thus we have a label set that is close in size to the one from the third level of the MIPS hierarchy. The “exact match” method then simply counts the number of correct labels, just as we did for a fixed level of the MIPS hierarchy. We see similar improvements for GO annotation as we did for MIPS annotation; detailed results for accuracy and F1 score for all four methods appear in (Figure S3).

However, note that this exact match evaluation gives no credit when we label a protein with an incorrect child that still has many ancestor terms in common with the correct label. Thus, for a more fair evaluation of the predictions that takes into account hierarchical relations among the GO labels, we also use the functional path method of Deng et al. [27]. This presents a plausible way to give partial credit when a node is labeled partially correctly, in the sense that the lowest depth child label assigned to the node and the correct label of the node are different, but if we consider the path in the ontology from the root to these two labels, there are ancestor labels these two functional paths have in common. We use exactly the functional path method of [27] to calculate precision and recall values for each protein, and then overall precision and recall values are averaged over all the proteins. We calculated both alternative ways to count functional paths overlap presented in Deng et al. [27], one that simply counts the number of nodes that appear jointly in sets of known and predicted functional annotations (which we will call the overlap counting method) and the other which takes into account at what depth the functional paths from both sets diverge (which we will call the overlap depth method).

**Figure 6:**
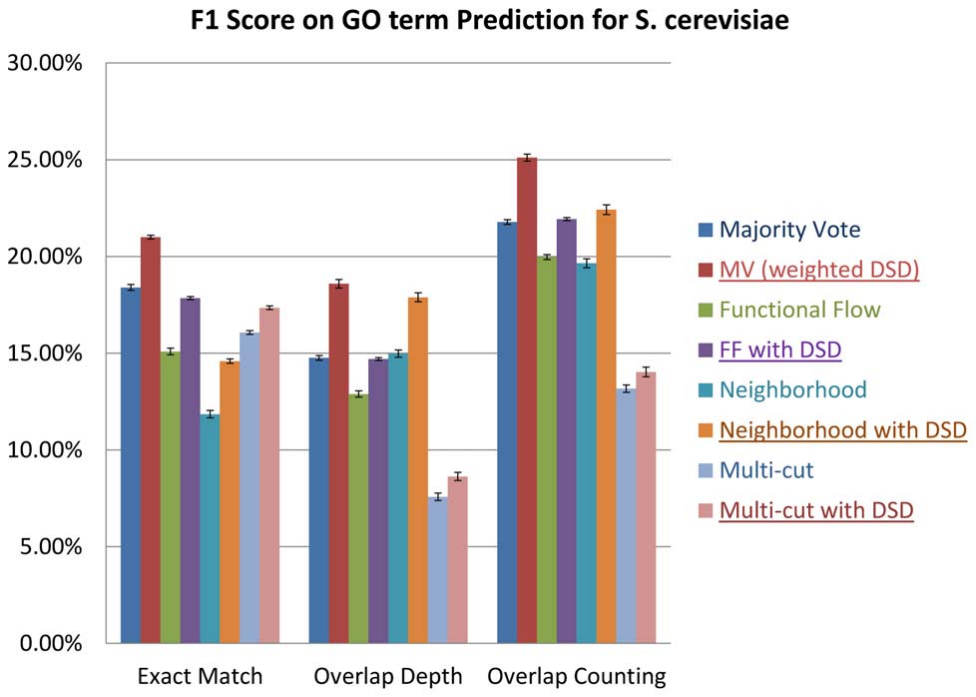
Improvement on F1 Score for DSD using three evaluation methods: exact match, overlap depth and overlap counting, on informative GO terms for the four algorithms for *S. cerevisiae* in 10 runs of 2-fold cross validation.

Setting 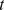, the neighborhood threshold for DSD majority voting anywhere in the range starting from 2, the DSD version of majority voting improves precision and recall simultaneously, as compared to original majority voting, regardless of the method used for counting overlaps. For example at 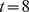, we get precision = 67.3% ±0.3% and recall  = 40.3% ±0.2% compared to precision  = 59.6% ±0.4% and recall  = 34.3% ±0.4% for the overlap counting method, and we get precision  = 61.1% ±0.3% and recall  = 27.3% ±0.1% compared to precision  = 52.9% ±0.5% and recall  = 20.5% ±0.4% for the overlap depth method, for 2-fold cross validation on the yeast PPI network. In fact, we find that all DSD versions of the four algorithms perform better than their ordinary distance versions, regardless of the methods used for keeping track of overlaps. Figure 6 shows the improvements in F1 scores (setting 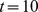 and 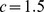 as before) over all three methods of counting the overlaps. Thus DSD is improving functional annotation also for GO annotation.

## Discussion

Our function prediction results demonstrate the utility of defining and using a fine-grained distance measure that is specifically tailored to the subject application domain. For the PPI network, the observation that shortest-paths that go through hubs are less informative led to the particular design of the He () measure incorporated into the DSD definition. The He () measure has connections to other diffusion-based measures previously proposed [23]; however, using He () globally across all nodes, and comparing such vectors via L1 norm is entirely new and enables DSD to capture truly global properties of network topologies.

**Figure 7:**
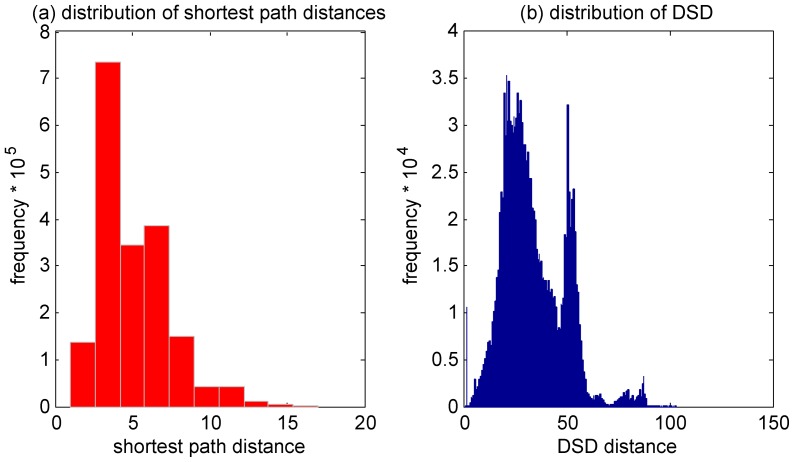
Shortest path distance and DSD distribution for *S. pombe.*

We have shown that substituting DSD for ordinary shortest path distance results in dramatic improvements when using network information to predict protein function for the *S. cerevisiae* PPI network. We expected that similar improvements would hold for the PPI-networks of other organisms as well, though the sparseness of current experimental known functional annotation and the number of PPI interactions currently known for other organisms means that we would not yet expect absolute levels of accuracy that are comparable to those achieved for *S. cervesiae.* We tested this intuition by also considering what is known of the PPI network of a different, less-well annotated yeast species, *S. pombe.* Based on the interactions in BIOGRID [20] version 3.2.102, the largest connected component has 1925 nodes and 4874 edges. Thus compared to the *S. cerevisiae* network, a much smaller and sparser subset of the PPI network is known. Figure 7 shows the shortest path and DSD distance distribution of this network. As expected, it is low-diameter but not yet as low-diameter as the *S. cerevisiae* network, and the DSD distribution is more spread out, but not yet as smooth as for the *S. cerevisiae* network. We would predict that the distribution would start looking more like *S. cerevisiae* as more of the network becomes known. We found no reliable MIPS annotation for *S. pombe,* so we instead used GO annotation, which we downloaded from the Pombase database, data version 2013-07-01 (http://www.pombase.org). We extract “biological process” GO terms only and remove GO terms annotated with evidence codes “IEA”, “IPI” and “RCA”, leaving 85 *informative* (see GO results section above, for definition) GO terms annotating 1722 out of 1925 proteins, with a total number of 5818 annotations. We did the “exact match” version of GO evaluation for 10 runs of cross-validation, and looked at the difference in performance for majority vote and functional flow methods using ordinary shortest path distance and DSD. We see similar improvements using DSD as with *S. cervisiae*; results appear in (Figure S4).

We present a simple to use, freely-available tool that given any PPI network will produce the DSD values between each pair of proteins, either as a webserver or for download from http://dsd.cs.tufts.edu/. This tool both allows the calculation of 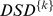 for some chosen 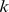, and, as in the results presented in this paper, for DSD in the limit. In fact, we also tried 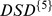 in place of DSD for all the methods in this paper and results were already quite similar to DSD in the limit. We suggest based on the dramatic improvements for the *S. cerevisiae* PPI network that DSD values be used in place of ordinary distance when using network information to predict protein function, either using the PPI network alone, or as part of a modern integrative function prediction method that includes data from a variety of sources beyond the PPI network, such as sequence information, genetic interaction, structural information or expression data [8], [13]–[15].

## Acknowledgments

Crovella and Cowen thank the Institute for Mathematics and its Applications for inviting them to their “Network Links: Connecting Social, Communication, and Biological Network Analysis” workshop in March 2012, where we learned about each others' recent work and began the collaboration that resulted in this paper. We thank Andrew Gallant for helping review the DSD code.

## Supporting Information

Figure S1
**Improvement of mean Accuracy and F1 Score for DSD at different neighborhood thresholds for the** 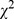 **neighborhood algorithm in 10-runs of 2-fold cross validation (with standard deviation error bars).**
(TIF)

Figure S2 **Improvement of mean Accuracy and F1 Score for DSD at different neighborhood thresholds for the multi-way cut algorithm in 10-runs of 2-fold cross validation (with standard deviation error bars).**
(TIF)

Figure S3 **Improvement of mean Accuracy and F1 Score for DSD at different neighborhood thresholds for all four methods in 10-runs of 2-fold cross validation (with standard deviation error bars) using the GO catagories (using the method that gives wcredit only for exact matches to each GO term).**
(TIF)

Figure S4 **Performance evalutaion for GO term prediction on the** ***S. pombe*** **network. (a) Comparison of mean F1 score of DSD and non-DSD majority vote (setting t = 10) and functional flow (setting c = 1.5) algorithms using all three methods of counting GO term matches; (b1,b2) Comparison of mean accuracy and F1 Score under the exact match method for different settings of the parameters t and c, over 10 runs of 2-fold cross validation (with standard deviation error bars).**
(TIF)
