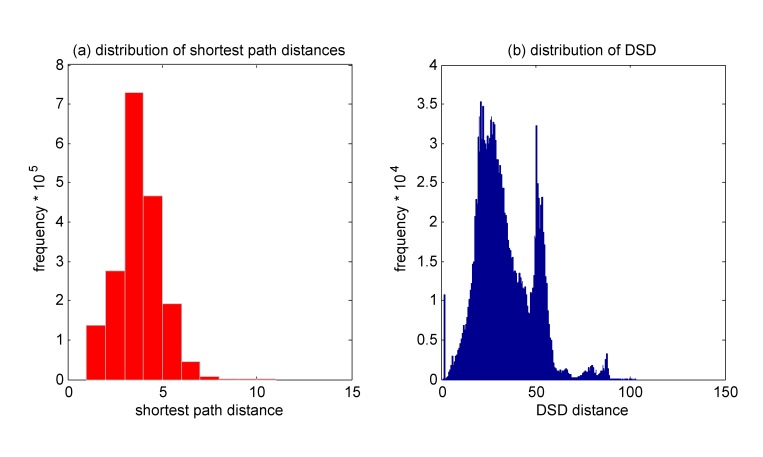# Correction: Going the Distance for Protein Function Prediction: A New Distance Metric for Protein Interaction Networks

**DOI:** 10.1371/annotation/343bf260-f6ff-48a2-93b2-3cc79af518a9

**Published:** 2013-10-30

**Authors:** Mengfei Cao, Hao Zhang, Jisoo Park, Noah M. Daniels, Mark E. Crovella, Lenore J. Cowen, Benjamin Hescott

Due to an error introduced in the production process, the x-axes in the first panels of Figure 1 and Figure 7 are not formatted correctly.

The correct Figure 1 can be viewed here: 

**Figure:**
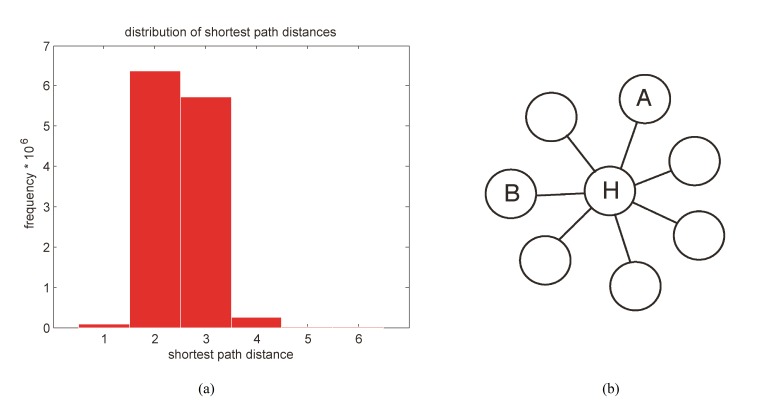


The correct Figure 7 can be viewed here: 

**Figure:**