# Supplementary figures and images for: Going the Distance for Protein Function Prediction: A New Distance Metric for Protein Interaction Networks
Source: PLoS One. 2013 Oct 23;8(10):e76339. doi: 10.1371/journal.pone.0076339 (PMC3806810; doi:10.1371/journal.pone.0076339)

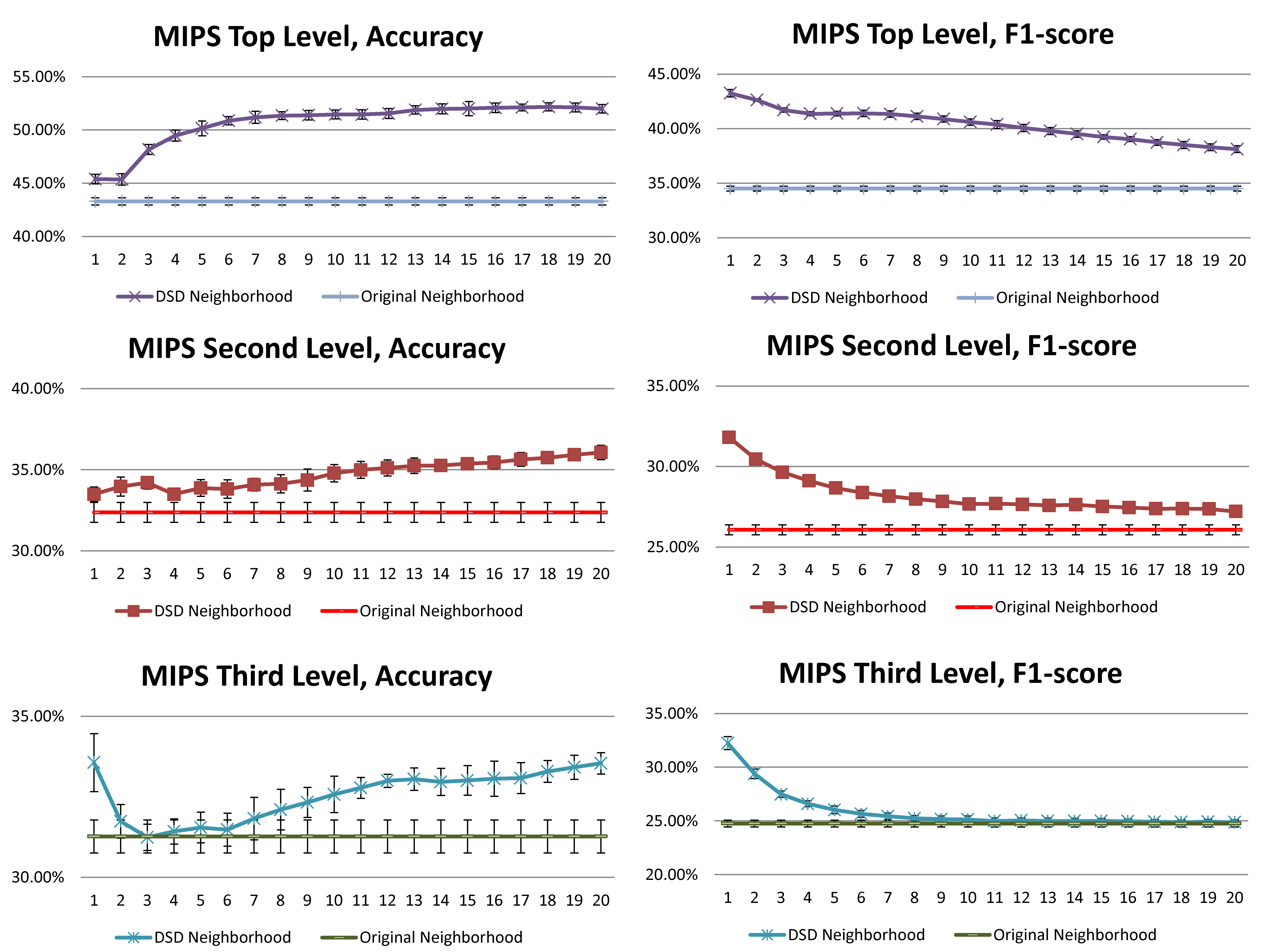

Supplement: Figure S1 — Improvement of mean Accuracy and F1 Score for DSD at different neighborhood thresholds for the neighborhood algorithm in 10-runs of 2-fold cross validation (with standard deviation error bars). (TIF) [file pone.0076339.s001.tif]

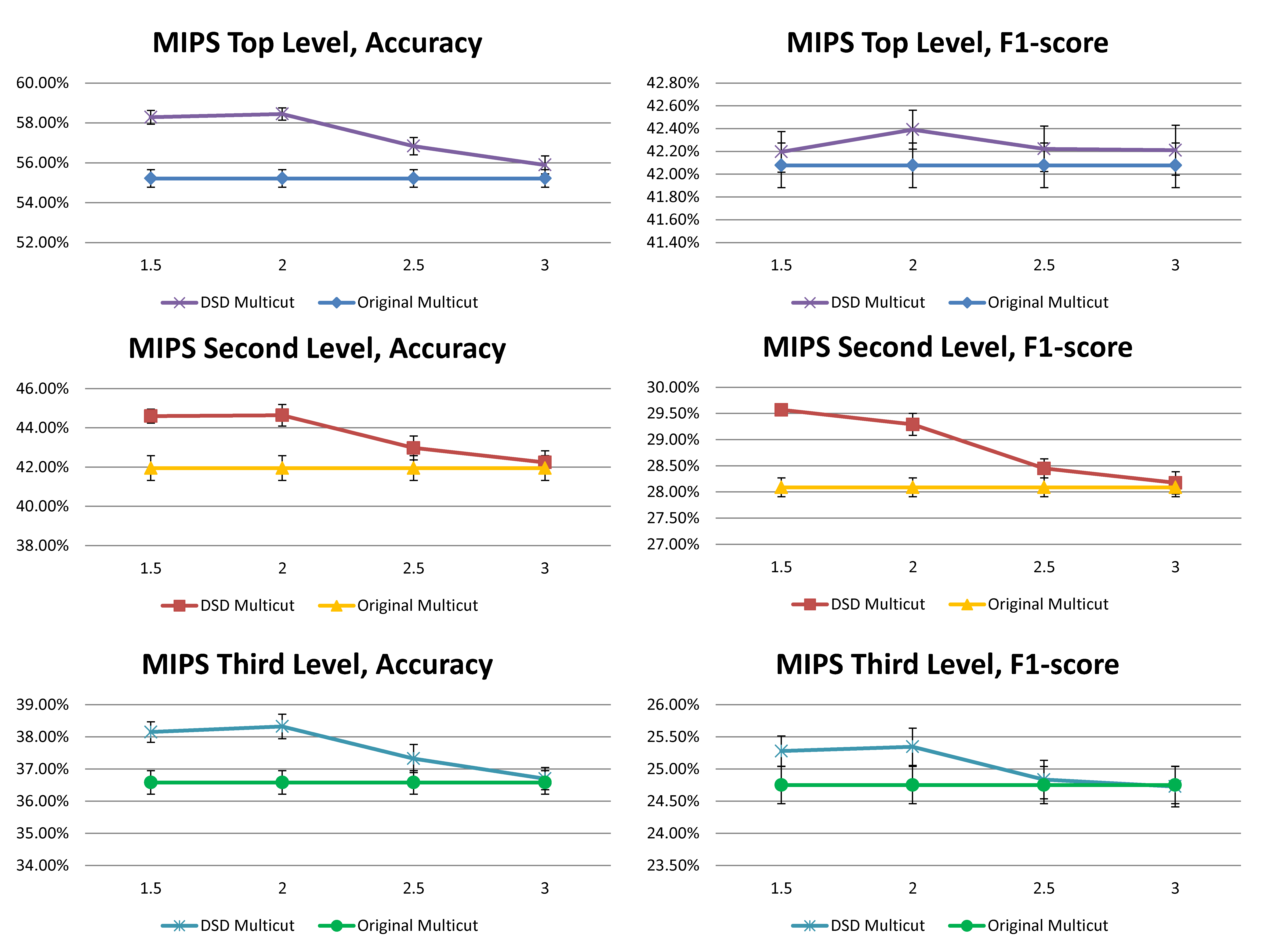

Supplement: Figure S2 — Improvement of mean Accuracy and F1 Score for DSD at different neighborhood thresholds for the multi-way cut algorithm in 10-runs of 2-fold cross validation (with standard deviation error bars). (TIF) [file pone.0076339.s002.tif]

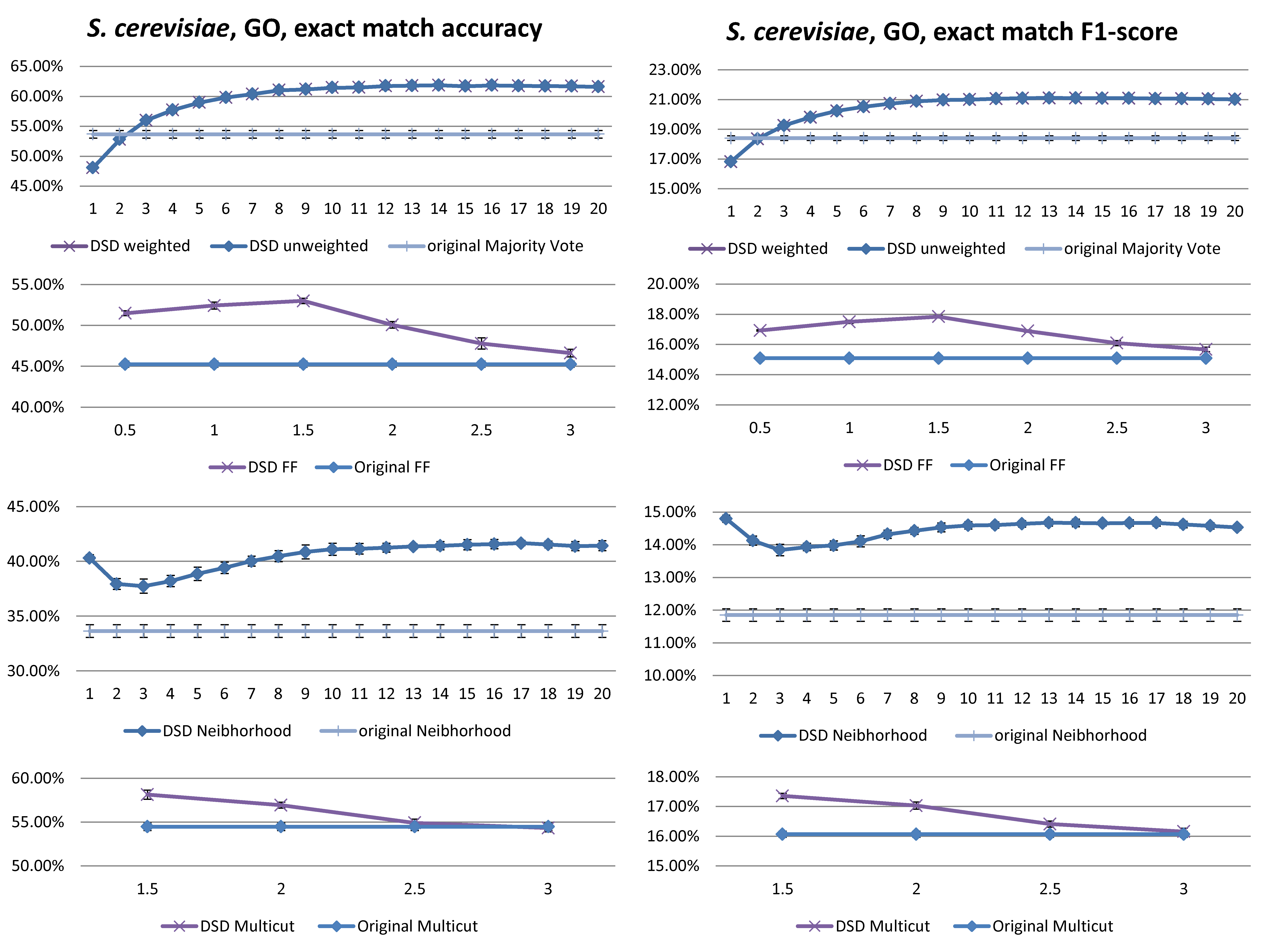

Supplement: Figure S3 — Improvement of mean Accuracy and F1 Score for DSD at different neighborhood thresholds for all four methods in 10-runs of 2-fold cross validation (with standard deviation error bars) using the GO catagories (using the method that gives wcredit only for exact matches to each GO term). (TIF) [file pone.0076339.s003.tif]

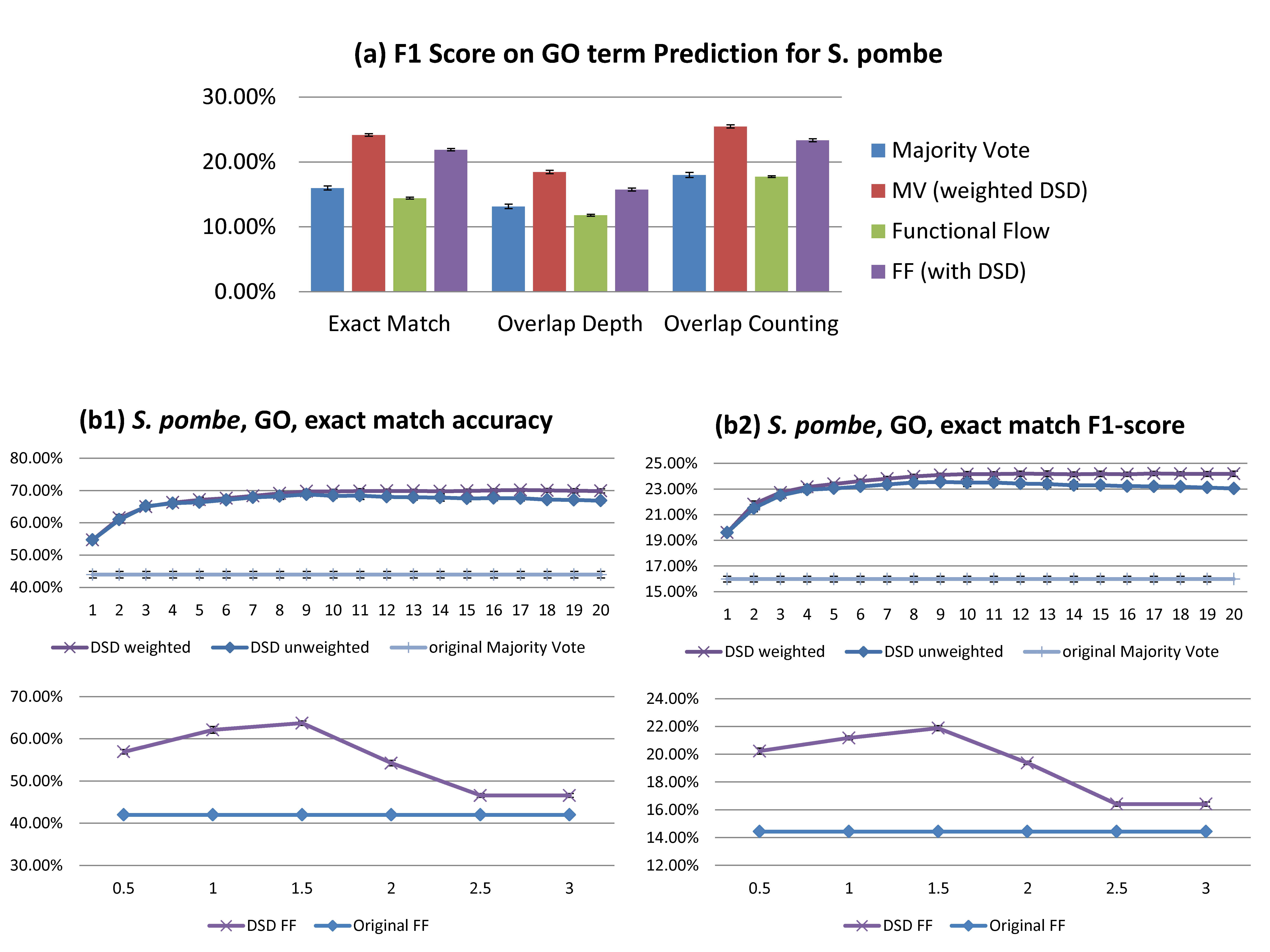

Supplement: Figure S4 — Performance evalutaion for GO term prediction on the S. pombe network. (a) Comparison of mean F1 score of DSD and non-DSD majority vote (setting t = 10) and functional flow (setting c = 1.5) algorithms using all three methods of counting GO term matches; (b1,b2) Comparison of mean accuracy and F1 Score under the exact match method for different settings of the parameters t and c, over 10 runs of 2-fold cross validation (with standard deviation error bars). (TIF) [file pone.0076339.s004.tif]
